# Editorial Correspondence

**Published:** 1883-01

**Authors:** 


### Editorial Correspondence.

Philadelphia, December 16, 1882.

Dear Journal—In addition to the hospitals mentioned in the last letter, there are several which are well worthy of a visit. Pre-eminent among them is the old "Pennsylvania," a mention of which will revive the thoughts of youth in many an old veteran in our ranks who walked its wards in his college days. Established one hundred and thirty-four years ago, it has, from its earliest days, freely opened its doors to the student; a wise management recognizing that in connection with the proper treatment of the sick in a hospital, one of the most beneficent uses of such an institution is the aid which it can and ought to give in propagating a wise system of medical education. The hospital, though in the centre of the business part of the city, is located in the middle of a large square, surrounded by a noble girdle of trees and open to the light and air on every side. Knowing that the main building had been erected more than a century ago, I was surprised to find its wards so admirably constructed and ventilated. The present system of ventilation is of recent adoption, but I was informed that the principal wards remain as originally constructed. A feature of the hospital is a very excellent medical library, which, for many years, was the largest and best in Philadelphia; now, however, that of the College of Physicians and Surgeons is the great library of the city. Connected with the hospital by a corridor is an octagonal building, of brick, capable of comfortably accommodating five hundred students. Clinics are held here every Wednesday and Saturday, from 10 to 12 A. M.; while at Jefferson Hospital, which is but a few blocks away, the clinics are from 12.30 to 2 P. M. The same arrangement exists at the "Philadelphia" and "University" Hospitals, so that on every clinic day one is free to choose between the four great hospitals, attending any two of them as the reputation of the clinician may attract.

Another great hospital, well worthy of a visit, though too far away from the colleges to be utilized for clinical instruction, is the "Episcopal" Hospital. Situated near the railroads and large manufacturing concerns, it is especially rich in surgical cases. The buildings, erected at a cost of about $350,000, consist of a central building, to which a fine chapel is connected, and two large wings. The wards are most bright and cheerful, particularly the children's ward, the walls of which are polychromed and handsomely illuminated with simple hymns and verses easily within a child's comprehension. As we entered the ward, a pretty picture presented itself of a charming young lady visitor, seated between two beds, in which lay two little children, whose wan faces showed a sad experience of suffering, but now lit up with interest and pleasure, while around the foot of the bed had gathered a little audience, some with crutches, one in a rolling chair, but all absorbed in some simple story which the young girl was reading to them. If any of my readers are familiar with the pitiable spectacles presented in the children's wards, at the old "Philadelphia Hospital," the rounds of which I had been in the habit of making, they will not wonder that I was struck with the contrast. The ventilation of the entire building is admirable, and, indeed, in all its appointments it appears to be a model hospital. I was particularly surprised by the statement of Dr. Knight, the superintendent, that they had not a single paying patient, the hospital being supported entirely by the voluntary offerings of the churchmen of the diocese, although the annual cost of its maintenance approximates $50,000. Notwithstanding its objectionable name, its catholicity is manifested by an inscription on its wall which testifies that it is open to all sufferers without distinction as to creed, color or nationality.

To-day, at the "Pennsylvania," R. J. Levis showed the successful result of plastic operations on a man whose face, neck and breast had been severely burned, resulting in such cicatricial contraction as to prevent the elevation of the chin from the breast; his face, also, had been terribly disfigured. These deformities had been pretty well relieved by several operations, but there remained marked ectropion of the lower lids. As the face was covered with cicatricial tissues, an attempt was made to supply the integument necessary, by the insertion of a piece of skin from a rabbit. Levis is a brilliant operator and a good lecturer. Among the surgeons in active practice here, though, there is no one, I think, who competes with Agnew for the first place. His clinics are always largely attended, and it is a pleasure to see him operate; there is no display, no haste, yet his work is done with rapidity and effectiveness. His lectures are delivered in the same quiet way. I have attended a few of them; but as a lecturer he is not, I think, the equal of Moore, of the Buffalo College; the students, however, consider him an admirable teacher, and they are good judges.

I have no hesitation in speaking of Pepper as the best clinical lecturer I have ever heard. I am glad to be able to send you a report of one of his lectures, taken verbatim by an accomplished stenographer. Very few teachers, indeed, have such a command of language as to be able to endure an exact reproduction of their words in cold print.

All of the professors here deliver their lectures without notes; in some departments not an easy thing to do, but certainly the most effective way in teaching. There is, indeed, a spirit of youth and progress in everything about the old university, due, I believe, to the number of comparatively young men in its corps of teachers. Pepper, Goodell, H. C. Wood, Tyson, Duhring, are all men well under 50, and connected with the university as demonstrators, lecturers, etc., are a great number of younger men, some of whom already press the professors pretty closely as to their local reputation for ability.

The pathological laboratory is presided over by Dr. Formad. Most of the readers of the JOURNAL are familiar with his name through the admirable researches on the micrococci of diphtheria, accomplished conjointly with H. C. Wood, and published by the National Board of Health. His recent article read before the Philadelphia County Medical Society, in refutation of Koch's theory as to the etiology of tuberculosis, has also deservedly attracted a good deal of attention. In that paper he claims to have positively proved that true tuberculosis can be produced without the agency of the bacilli tuberculosis. He does not deny their presence, but asserts that they are secondary, the tubercular tissue serving merely as a nidus for their growth. Ergo phthisis is not a specific infectious disease, but may occur in man or animals having a scrofulous diathesis. Such animals will become tubercular from any injury resulting in inflammation or from repeated injuries. That scrofulosis, and subsequently tuberculosis, can be induced artificially in animals normally not possessed of this condition, e. g., non-scrofulous animals, the cat, for instance, by being kept in confinement and poorly nourished acquire a scrofulous anatomy and will then succumb to tuberculosis if any inflammatory process is set up. No specific action of bacilli is required. The inoculation of perfectly innocuous foreign materials, such as sand, or pieces of glass, or metal accomplishing the result.

I asked Formad if his present position did not militate somewhat against the result of his researches as to a specific micrococcus in diphtheria. The candor of his reply was refreshing: "What if it does? I have often been mistaken; I have to tell the truth as I find it; if I don't, somebody else will." The question is one of the greatest importance, and if he can, in this case, maintain his position, it will reflect no little honor on American pathologists, and will be another notable example of the scientific progress of this country. We have, perhaps, all been accustomed to depreciate the value of work accomplished in our own laboratories in comparison with European pathological researches, but in this, as already in other departments of medicine, we must eventually come to the fore.

H. C. Wood, whose opinion upon such a subject is of the highest value, considers Koch's theories as highly improbable and *opposed* by the well-known *clinical* facts of the disease, while another authority asserts that a contagious materies morbi is in a high degree probable, because *confirmed* by *clinical* experience as well as experimental study. With these two directly contrary expressions of opinion I will drop the subject, convinced that those of us who have accepted Koch's theory, are not going to give up our bacilli without more evidence than we yet have, that they are not the little demons we suspect them to be.

I had the pleasure of hearing the first lecture, of a course of three, to be delivered by Dr. Austin Flint, under the auspices of the Philadelphia County Medical Society. His subject was "The Physical Exploration of the Lungs by Means of Auscultation and Percussion." Much curiosity was manifested before the first lecture as to what the distinguished clinician could possibly say on this subject, which was not already well known. The hall, on the evening appointed, was filled to overflowing, indeed many could not obtain admittance. This lecture was limited to a consideration of "the true mode of study and its requirements as regards auscultation and percussion, and the signs obtained by percussion." Although the lecturer presented no ideas other than those already universally accepted and so well taught in his "Manual of Percussion," etc., still it came with such a freshness and vigor from the lips of the distinguished author that it was listened to with apparent interest and pleasure by his audience, though many of them were themselves teachers of no mean rank. One novelty, however, he did give us, and that was the employment of loaves of brown bread to demonstrate the signs obtained by percussion.

Certainly the sounds obtained by percussion with a hammer or a loaf of bread enveloped in a towel are strikingly like the normal pulmonary resonance. Absence of resonance, or flatness, was illustrated by percussing on a loaf of bread, one-half of which had been dipped in a solution of gelatin. The "flatness" produced by the filling up of the vesicles of the bread on one side was in marked contrast with the normal resonance on the other. Diminished resonance, or dullness, was produced by introducing some sticks of candy into the bread. Tympanitic and amphoric resonance was closely simulated by forming cavities in the loaf under various conditions. Flint still commends the hammer and pleximeter which here is rarely used.

I cannot take my leave of Philadelphia without referring to the hospitality and cordiality with which the medical stranger is received by the profession here. There is no end of doctors; they are decidedly more numerous than in Buffalo; but, so far as my limited acquaintance went, they are not only well educated and hard workers, but a more genial and pleasant body of men, as met professionally at the associations, or hospitals, or in society, it would be hard to find.

